# Learning Autonomy and Group Cohesion in Clinical Simulation: A Quasi-Experimental Comparison of Two Training Approaches

**DOI:** 10.3390/nursrep16060199

**Published:** 2026-06-11

**Authors:** José Manuel García-Álvarez, Alfonso García-Sánchez, José Luis Díaz-Agea

**Affiliations:** 1 Health Sciences PhD Program, Catholic University of Murcia (UCAM), Campus de los Jerónimos nº 135, 30107 Murcia, Spain; 2 Faculty of Nursing, Catholic University of Murcia (UCAM), Campus de los Jerónimos nº 135, 30107 Murcia, Spain; agarcia@ucam.edu; 3 Faculty of Nursing, University of Murcia, Campus de Ciencias de la Salud, Edificio LAIB/Departamental, El Palmar-Murcia, 30120 Murcia, Spain; agea@um.es

**Keywords:** group cohesion, clinical simulation methodologies, nursing education, quasi-experimental study

## Abstract

**Background/Objectives**: Given the complexity of current healthcare, working in highly cohesive teams is essential. Clinical simulation can promote group cohesion among healthcare teams. There are different learning methodologies in simulated environments capable of developing group cohesion in healthcare teams. The objective of this study was to compare group cohesion outcomes between two clinical simulation learning models—self-directed (Self-Learning Methodology in Simulated Environments, MAES©) and non-self-directed (Simulation-Based Learning, SBL)—in nursing student teams. **Methods**: A quasi-experimental pre–post study with a control group was conducted among 311 fourth-year nursing students from two Spanish universities. The primary outcome was group cohesion, assessed using the Spanish short version of the Group Environment Questionnaire (GEQ) total score and its dimensions. The questionnaire was administered twice, before and after participation in clinical simulation sessions. The experimental group used the MAES© methodology, while the control group used SBL. Between-group differences were analyzed using analysis of covariance (ANCOVA), adjusting post-intervention scores for baseline values. Within-group pre-post changes were explored as a secondary analysis using the Wilcoxon signed-rank test. **Results**: No significant differences in baseline group cohesion were found. After the intervention, both methodologies were associated with improvements in group cohesion over time. Adjusted analyses (ANCOVA) showed statistically significant between-group differences favoring MAES© across all dimensions, with small-to-medium effect sizes (ηp^2^ = 0.036–0.138). **Conclusions**: Both simulation methodologies were associated with improvements in group cohesion among nursing students. Adjusted between-group differences were observed across all dimensions, associated with higher adjusted scores in the MAES© group. However, given the non-randomized design these findings should be interpreted as associations rather than evidence of causality. Further randomized controlled trials are needed to confirm these results.

## 1. Introduction

The increasing complexity of today’s healthcare system requires highly cohesive work teams capable of delivering high-quality and efficient healthcare [1,2]. Technological progress and the need to maintain continuity of care demonstrate that individual work is not sufficient to comprehensively address patients’ needs [3,4,5,6]. Teamwork has been associated with more efficient use of available resources, improved performance, reduced work-related stress, and increased professional satisfaction [7,8].

Healthcare team cohesion refers to the set of processes that keep a team united while carrying out its care activities, promoting coordinated actions among team members to achieve shared goals. Group cohesion has a component related to accomplishing common objectives, task cohesion, and another component focused on the development of interpersonal bonds among team members, social cohesion. These two components can be considered both individually and collectively to describe four fundamental dimensions of group cohesion: Group Integration—Task (GI-T), understood as the degree of collective commitment to common goals; Group Integration—Social (GI-S), which reflects the sense of belonging and social connection within the team; Individual Attraction to Group—Task (ATG-T), referring to each member’s personal motivation toward common objectives; and Individual Attraction to Group—Social (ATG-S), which pertains to individual interest in the team’s social relationships [9,10,11,12,13,14].

These four dimensions of group cohesion can be empirically assessed using the Group Environment Questionnaire (GEQ) [15,16]. In the Spanish context, there are several adaptations of this instrument applied to different populations, such as athletes [17], non-healthcare university students [18], and nursing students [19]. The short version validated with nursing students undergoing clinical simulation training has shown adequate levels of internal consistency, homogeneity, and temporal stability. Additionally, the factor analyses supported the original four-dimension structure of the instrument. Therefore, this short Spanish version of the GEQ can be considered a reliable and appropriate tool for evaluating group cohesion in nursing student teams in simulated environments [19].

For healthcare teams to function effectively, it is important that professionals develop interpersonal and cognitive skills (non-technical skills) that complement their clinical knowledge (technical skills). While technical skills focus on the correct execution of clinical procedures, non-technical skills include the abilities that facilitate collaboration, decision-making, and safety in professional practice. These non-technical skills include clear communication, task coordination, mutual trust, and commitment to the team. The development of these non-technical skills has been associated with improved team cohesion and the achievement of shared objectives, especially in environments where workload is high and emotional involvement—both from patients and professionals—is intense [20,21,22,23,24].

Clinical simulation has been widely used as a tool for developing the non-technical skills essential to improving group dynamics among teams of healthcare students and professionals. It is an educational methodology designed to facilitate experiential learning through the practical recreation of realistic clinical situations using simulated patients. Through scenarios that replicate real care settings, participants face situations that require teamwork, which may promote learning and the development of both technical and non-technical skills. This training approach has been associated with improvements in situational awareness, resource use, decision-making, and communication. In addition, clinical simulation has been associated with increased mutual trust, respect, collaboration, commitment, and leadership within the team [20,21,25,26].

Each simulation session is organized into three phases: the prebriefing phase, during which learning objectives and participant roles are defined; the simulation phase, in which students perform patient care tasks under realistic conditions; and the debriefing phase, which allows reflection on the simulated activity and consolidation of learning [27,28]. Through cycles of simulation and guided reflection, students may develop essential skills and experience shared challenges that can support coordination, trust, and group cohesion [20,21,22,25,26,29,30,31,32].

The implementation of clinical simulation is not only associated with improved teamwork and cohesion within healthcare student teams [33], but it may also help reduce stress, increase job satisfaction, and boost motivation among professionals [34,35,36]. These benefits may be associated with improvements in the efficiency and quality of patient care, supporting the relevance of clinical simulation as an educational strategy in healthcare team training.

Traditional instructor-led clinical simulation, known as Simulation-Based Learning (SBL), has been widely used and associated with the development of both technical and non-technical skills, as it provides healthcare students and professionals with opportunities to face realistic scenarios in a safe environment. However, this methodology depends on the presence of an instructor to guide the simulation exercises toward pre-established learning objectives and may offer fewer opportunities for learner autonomy and shared decision-making during the learning process [37,38,39,40].

For this type of learning to be effective and maximize learning benefits, it is generally recommended that simulated scenarios replicate reality as closely as possible. This typically involves careful planning, adequate infrastructure, and appropriate training for instructors guiding students through the simulation experience. Additionally, to achieve the intended competencies, learning objectives should be adapted to the students’ prior level of preparation and be clear, precise, and realistic. Scenario design should be developed gradually and follow a structured approach so that educational objectives remain central throughout the entire simulation [41,42,43,44].

The Self-Learning Methodology in Simulated Environments (MAES©) is an educational strategy that integrates clinical simulation with self-directed learning through teamwork. It integrates complementary learning models, such as self-directed learning, problem-based learning, collaborative learning, and peer-to-peer learning. This approach is designed to enable students to plan, execute, and evaluate their own learning process, as well as to support autonomy in selecting competencies they wish to develop. MAES© sessions are conducted progressively and interactively, combining in-person and remote activities across several stages: team formation, selection of the topic and problem to solve, identification of competencies and prior knowledge, design of the simulated scenario, execution of the simulation, and final debriefing. Overall, this methodology has been associated with the development of both technical and non-technical skills in healthcare settings [45,46,47,48], and with improvements in students’ critical thinking, motivation, and satisfaction [49,50,51].

The MAES© methodology may support the development of essential non-technical skills, such as communication, leadership, decision-making, teamwork, and stress management, which may contribute to students’ performance and comprehensive training in clinical settings. Additionally, by promoting situational awareness, coordination, and group engagement, this methodology may contribute to enhancing team cohesion, fostering cooperation, shared responsibility, and a sense of collective identity [45,46,47,48].

The MAES© methodology, based on autonomous and self-directed learning, is proposed as an alternative to traditional instructor-led clinical simulation, the SBL methodology. However, there is limited evidence directly comparing the effects of these simulation approaches on group cohesion, particularly in nursing student teams. Furthermore, the mechanisms through which different methodologies may influence cohesion-related dimensions remain insufficiently explored. The results of this study may contribute to the evidence base on simulation-based education and inform training approaches aimed at developing cohesive and effective healthcare teams.

Previous studies have reported positive associations between clinical simulation and teamwork [32], as well as group cohesion among nursing students [33]. However, little is known about whether different simulation approaches—specifically self-directed and instructor-directed formats—have distinct effects on group cohesion.

In this context, it was hypothesized that, given its emphasis on autonomy, collaboration, and shared decision-making, the MAES© methodology may be associated with greater improvements in group cohesion scores compared with SBL.

The objective of this study was to compare group cohesion outcomes between two clinical simulation learning models—self-directed (Self-Learning Methodology in Simulated Environments, MAES©) and non-self-directed (Simulation-Based Learning, SBL)—in nursing student teams.

## 2. Materials and Methods

### 2.1. Ethics Statement

This study was conducted in accordance with the ethical principles established by the Declaration of Helsinki [52] and was approved by the Ethics Committee of the Catholic University of Murcia (UCAM).

All students were provided with detailed information about the study, emphasizing that although participation was part of the regular curriculum, participation in the research component was entirely voluntary and would not affect their academic evaluation. Written informed consent was obtained from all participants, and data confidentiality was ensured through the use of encrypted databases and restricted access to research personnel.

### 2.2. Study Design

A quasi-experimental study with a pre-post design and a control group was conducted to evaluate changes in group cohesion associated with clinical simulation using two methodologies: self-directed (MAES©) and instructor-guided (SBL). The experimental group used the MAES© methodology, while the control group used SBL.

The study followed the TREND (Transparent Reporting of Evaluations with Non-Randomized Designs) reporting guidelines for non-randomized interventions [53]. Potential selection bias was considered in the interpretation of the results.

### 2.3. Subjects and Scope of Study

The study included fourth-year undergraduate nursing students from the Catholic University of Murcia (UCAM) and the University of Murcia (UMU), who participated in clinical simulation sessions between October 2023 and July 2024. All participants had prior experience in simulated environments, which may have helped reduce initial variability between groups and facilitated the interpretation of observed differences between groups.

### 2.4. Sample Selection

The study included fourth-year undergraduate nursing students from the participating universities who attended all clinical simulation sessions and agreed to participate voluntarily. All students who participated in clinical simulation during the study period were included, encompassing all eligible students available at that time.

Participants were selected through non-probabilistic convenience sampling, taking into account the methodology used at each university. Accordingly, students from UCAM comprised the experimental group, while those from UMU formed the control group. This allocation may introduce selection bias and potential institutional confounding related to differences in educational culture, faculty approach, and previous training experiences, which was considered in the interpretation of the results and may affect the generalizability of the findings.

No a priori sample size calculation was performed, as all eligible students available during the study period were included.

Only participants who completed both pre- and post-intervention assessments and attended all simulation sessions were included in the final analyses. Consequently, a complete-case analysis approach was used. The final sample included MAES© (n = 188) and SBL (n = 123) participants, with no losses to follow-up or exclusions due to missing data or non-attendance.

### 2.5. Measurement Instrument and Data Collection

For data collection, each participant completed the Spanish short version of the Group Environment Questionnaire (GEQ), previously validated in nursing students, at two time points: before the first simulation session (pre-intervention) and after the final session (post-intervention).

Between the two rounds of questionnaires, four weekly simulation sessions, each lasting four hours, were conducted weekly with groups of two to three students, who were already part of previously established academic simulation groups within their respective institutions. Each group prepared one scenario per session, either self-directed by the students or instructor-led, depending on the methodology used, which facilitated opportunities to practice complex technical and non-technical skills in the context of specialized care. The simulations were carried out using a high-fidelity simulator (SimMan 3G; Laerdal, Stavanger, Norway). After each scenario, a structured Plus/Delta debriefing session was conducted, facilitated by instructors trained in both methodologies, with a more directive role in SBL and a facilitative role in MAES©, aimed at identifying aspects to be maintained (Plus) and those requiring improvement (Delta).

Before the study began, all instructors received training in their assigned simulation method. The topics, objectives, and session lengths were the same for both groups, but the teaching style differed: Simulation-Based Learning (SBL) was more directive, while MAES© fostered a facilitative approach. Faculty members met regularly to ensure consistency across groups, so that the main difference between them lay in the simulation methodology itself.

The initial questionnaire included sociodemographic data and the abbreviated Spanish version of the Group Environment Questionnaire (GEQ), validated in nursing students in clinical simulation settings [19]. The final questionnaire included only the GEQ. The GEQ consists of 12 items with a five-point Likert-type response scale ranging from “strongly disagree” (1) to “strongly agree” (5). Items 1, 3, and 5 assess the Individual Attraction to Group—Social (ATG-S) dimension; items 2, 4, and 6 assess the Individual Attraction to Group—Task (ATG-T) dimension; items 7, 9, and 11 assess the Group Integration—Social (GI-S) dimension; and items 8, 10, and 12 assess the Group Integration—Task (GI-T) dimension (Table 1).

The variables analyzed included the simulation methodology (MAES©/SBL), age, gender, region of origin, previous academic qualifications, employment status, dimension scores, and the total instrument score.

### 2.6. Data Analysis

The data were entered into a database and subsequently analyzed using the statistical software SPSS v26^®^ for Windows (IBM Corp., Armonk, NY, USA). Post hoc sensitivity analyses were conducted using G*Power 3.1^®^ for Windows (Heinrich Heine University Düsseldorf, Düsseldorf, Germany) to estimate the minimum detectable effect size based on the final sample size.

For dichotomous or polytomous qualitative variables, frequencies and percentages were calculated. For quantitative variables, the mean was estimated as a measure of central tendency and the standard deviation (SD) as a measure of dispersion, with normality assessed using the Kolmogorov–Smirnov test. For ordinal qualitative variables, the median was calculated as a measure of central tendency and the interquartile range (IQR) as a measure of dispersion [54,55].

To evaluate associations among the variables analyzed, appropriate statistical tests were applied depending on the nature and distribution of the data. Statistical significance was set at *p* < 0.05 [56,57].

The primary outcome was perceived group cohesion at the individual level, measured using the short Spanish version of the Group Environment Questionnaire (GEQ). To assess the influence of each clinical simulation methodology (MAES© vs. SBL) on group cohesion, changes in dimension scores and the total GEQ score were analyzed between pre- and post-intervention measurements.

The primary analysis consisted of a comparison between groups using analysis of covariance (ANCOVA), adjusting post-intervention scores according to baseline values and calculating effect size using partial eta-square (ηp^2^) together with their 95% confidence intervals (95% CIs). Effect sizes were interpreted as small (0.01), medium (0.06), and large (0.14) [58,59,60].

The GEQ dimension scores, obtained using Likert-type items, were treated as approximately continuous variables, since composite scores are usually analyzed as interval measures. The ANCOVA assumptions were assessed using residual diagnostics, normality tests, homogeneity of variance tests, and visual inspection of scatter plots to evaluate the linear relationship between baseline and post-intervention scores. The homogeneity of regression slopes was examined using the interaction test between baseline and group scores, and no significant interaction was found. Therefore, the ANCOVA assumptions were considered sufficiently met.

However, complete independence of observations cannot be assumed because students were nested within simulation groups and universities. This potential clustering effect should be considered when interpreting the findings. To assess the robustness of the results, a sensitivity ANCOVA adjusting for province of origin was also conducted.

Changes within each group between pre- and post-intervention measurements were analyzed as a secondary exploratory analysis using the Wilcoxon signed-rank test. Effect sizes were calculated using the Rosenthal correlation coefficient (r = |Z|/√N) with 95% confidence intervals and interpreted as small (<0.3), medium (0.3–0.49), large (0.5–0.69), and very large (≥0.7) [58,59,60,61,62].

These analyses were included to provide additional context, although they do not account for baseline differences between groups or other potential confounders, so the results should be interpreted cautiously. The main analysis was conducted at the individual level, treating each student independently and conceptualizing group cohesion as each participant’s perceived level of cohesion. Students were nested within simulation groups and universities, but multilevel models were not used. This was an exploratory study, and given the very small size of the simulation groups (2–3 students), multilevel modeling was not considered appropriate.

## 3. Results

**Figure 1:**
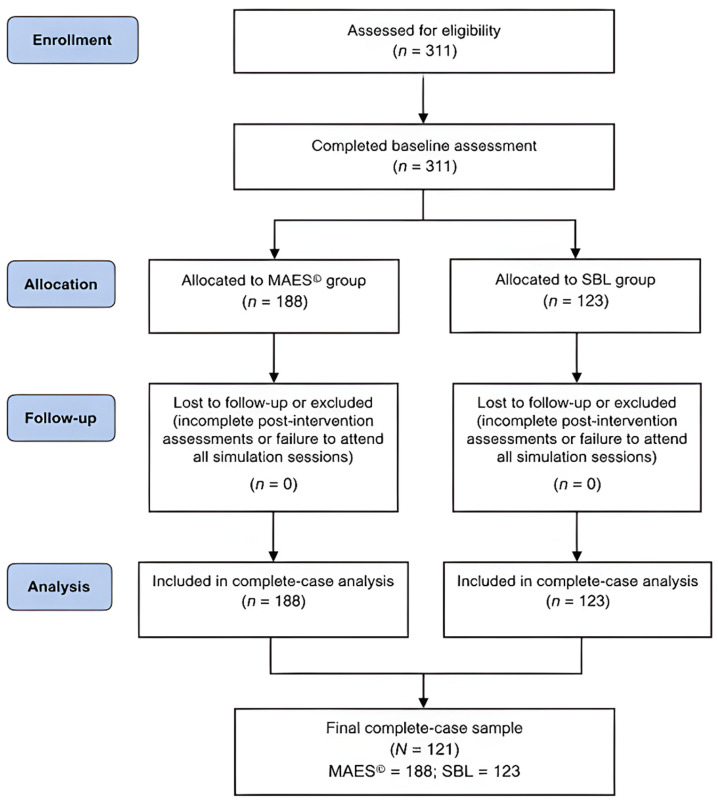
Participant flow diagram according to TREND guidelines.

Figure 1 presents the participant flow throughout the study according to TREND guidelines.

No significant differences were found between groups in age, gender, previous academic qualifications, or employment status. A significant difference was observed in the province of origin (*p* < 0.001), with a higher proportion of participants from Murcia in the SBL group (93.94%) compared with the MAES© group (56.38%) (Table 2).

A post hoc sensitivity power analysis indicated that the achieved sample size (N = 311; MAES© = 188, SBL = 123) provided 80% power to detect small-to-moderate between-group effects (ηp^2^ ≥ 0.03) at α = 0.05.

Table 3 presents descriptive statistics for the GEQ dimensions before and after the intervention in both groups. Overall, perceived group cohesion increased across all dimensions after the intervention. The MAES© group showed clearer improvements and less variability in post-intervention scores (IQR), while changes in the SBL group were smaller and less stable, particularly in the social cohesion dimensions.

The adjusted ANCOVA results showed significant between-group differences across all dimensions of group cohesion after the intervention. The strongest effect was observed in the Group Integration—Task dimension (GI-T; ηp^2^ = 0.138), which falls within the small-to-moderate range according to conventional benchmarks (Table 4). Sensitivity analyses adjusting for province of origin produced essentially the same pattern of results, with no meaningful changes in effect sizes or statistical significance.

In the within-group analysis, both groups showed statistically significant pre-post improvements across all dimensions (*p* < 0.001). Effect sizes were larger in the MAES© group for all dimensions, with the most pronounced differences observed in GI-T and the total score (Table 5).

## 4. Discussion

The results of this study showed that both the Self-Learning Methodology in Simulated Environments (MAES©) and Simulation-Based Learning (SBL) were associated with improved perceived group cohesion at the individual level. The primary inference of between-group differences is based on adjusted ANCOVA results controlling for baseline values, which consistently favored the MAES© group across all dimensions and showed higher post-intervention group cohesion scores.

Within-group analyses showed statistically significant pre–post improvements in both groups across all cohesion dimensions. Although these analyses were exploratory and descriptive and should not be interpreted as evidence of superiority because they do not account for baseline differences or potential confounding variables, effect sizes were consistently larger in the MAES© group across all dimensions and in the total score.

The adjusted between-group analyses showed that the largest difference associated with the MAES© methodology was observed in the Group Integration—Task (GI-T) dimension (ηp^2^ = 0.138), suggesting higher post-intervention task-oriented cohesion scores in the MAES© group after controlling for baseline values. Several studies have indicated that high task-oriented cohesion is associated with higher scores on team outcomes [4,5,8]. This finding is particularly relevant in healthcare settings, where a focus on shared clinical objectives represents a fundamental component of performance and patient safety [5,8,63]. More broadly, fostering autonomy has been identified in the literature as an important educational strategy to address the increasing complexity and demands of contemporary healthcare systems [64,65,66,67], and has also been associated with improved performance and engagement in simulation-based and MAES©-guided learning environments [49,65]. In this context, the present results may suggest a potential role for the MAES© methodology in supporting task-related aspects of cohesion.

Within-group analyses showed pre–post increases in total group cohesion scores in the MAES© group; however, these results are descriptive and should be interpreted with caution, as they do not adjust for baseline differences or confounding and do not support between-group comparisons. While the MAES© methodology was associated with larger within-group improvements in task-oriented cohesion, its association with social cohesion was more moderate but still meaningful. Specifically, the effect sizes for Individual Attraction to Group—Social (ATG-S) were large, whereas those for Group Integration—Social (GI-S) were medium, suggesting meaningful improvements in interpersonal bonding within the team. These findings suggest that, although improvements were more pronounced in task cohesion, positive changes in social cohesion and team dynamics were also observed, albeit to a lesser extent. Clinical simulation has been associated with the development of mutual trust, supportive relationships, open communication, and a sense of belonging that may extend beyond the simulated experience [68,69,70], potentially contributing to social cohesion [33]. In this context, the MAES© methodology may support these processes through its emphasis on teamwork and group identity.

The largest effect size observed in the Individual Attraction to Group—Task (ATG-T) dimension in the MAES© group was large, indicating a within-group improvement in individual engagement with group objectives. These results suggest that MAES© may be associated with increases in individual commitment to group tasks. However, the methodology appears to emphasize the achievement of objectives through cooperative work dynamics, prioritizing collective over individual performance [45,46,47,48].

The MAES© methodology, unlike the SBL methodology, is based on self-directed, collaborative, and peer-to-peer learning, allowing students to take an active role in their educational process, while the instructor acts as a facilitator [45,46,47,48]. Several characteristics of this approach may help to contextualize the observed differences in group cohesion, including the joint design of scenarios by each team and the shared definition of learning objectives. In addition, the use of structured collaborative tasks and constructive performance comparison may contribute to increased engagement and motivation within teams. These features may be associated with a sense of shared responsibility and participation in goal setting, which could in turn support team cohesion. Together, these elements may help create an environment that fosters communication, trust, and group identity, which could partially explain the higher adjusted cohesion scores observed in the MAES© group.

Overall, the findings of this study indicate that the MAES© methodology is associated with improvements in both task-oriented cohesion and social cohesion, achieving a larger effect size across all evaluated dimensions (ATG-S, ATG-T, GI-S, and GI-T). This pattern suggests that the approach is associated with higher individual commitment to group tasks and stronger interpersonal bonds and the sense of belonging within the team. The combination of cooperative work dynamics, autonomous learning, and guided reflection appears to create an environment conducive to simultaneously developing collective effectiveness and social cohesion. In healthcare settings, where coordination, mutual trust, and the ability to achieve shared clinical objectives are essential, implementing MAES© could contribute to training competent, resilient, and cohesive teams, reinforcing both the technical and social dimensions of professional performance.

Among the strengths of the study are the relatively large sample size, the use of a validated instrument for the clinical simulation context (short Spanish version of the GEQ), and a robust statistical approach, including the use of ANCOVA to adjust for baseline differences and the estimation of effect sizes. However, the study also has several limitations related to its non-randomized design and the use of convenience sampling, which should be considered when interpreting the findings.

An important potential source of confounding comes from the allocation of participants by university. Because all MAES© participants came from one institution and all SBL participants from another, the observed between-group differences could partly reflect institutional factors rather than the intervention itself. Relevant factors include differences in academic culture, prior exposure to collaborative learning, teaching practices, peer dynamics, and broader organizational contexts.

Although sensitivity analyses adjusting for province of origin yielded similar results, some residual institutional confounding cannot be ruled out. For this reason, the between-group differences cannot be attributed solely to the simulation methodology. Overall, the findings should therefore be interpreted with caution when considering causal inferences about the relative effectiveness of the two approaches.

A major limitation of this study is its quasi-experimental, non-randomized design, which limits the ability to draw causal inferences regarding the effects of the interventions on group cohesion. The absence of randomization also increases the risk of selection bias and residual confounding, which may have influenced the observed outcomes. Potential differences in institutional culture, pedagogical approaches, instructor characteristics, and students’ prior educational experiences between universities represent plausible alternative explanations for the observed outcomes. In addition, pre-existing academic group composition could also have affected cohesion outcomes independently of the intervention.

No a priori sample size calculation was conducted, as the study included all eligible students available during the study period, with the final sample size determined by feasibility rather than predefined recruitment targets. Furthermore, participants were nested within simulation groups and universities, and each simulation methodology was implemented in a different university, resulting in complete confounding between the intervention and the institutional context.

Although group cohesion is conceptually a group-level construct, the analyses were conducted at the individual level. As a result, it was not possible to disentangle the effects of the educational methodology from potential institutional influences, which should be taken into account when interpreting the findings. Another limitation is that the sample consisted exclusively of fourth-year nursing students from two Spanish universities, which limits generalizability to other academic years, healthcare disciplines, educational systems, or cultural contexts. Future multicenter studies including participants from different health professions and training levels would strengthen external validity.

Future research should employ randomized controlled trials to strengthen causal inference and reduce potential bias. This would also enhance the robustness and generalizability of the findings.

While province of origin was statistically controlled in the sensitivity analyses, other unmeasured confounders could have influenced the results. These include prior teamwork experience, individual personality traits, baseline motivation, and instructor-related factors such as teaching style or facilitation quality. Because these variables were not measured, their influence cannot be ruled out, which is an inherent limitation of quasi-experimental educational research.

Although baseline differences were partially adjusted using ANCOVA, some initial disparities between groups remained and may still have influenced the results. While statistical adjustment of baseline scores provided a more accurate estimation of the intervention effects, these differences should be better controlled in future studies, ideally through stronger design strategies that ensure baseline comparability between groups from the outset.

Another limitation is that group cohesion was assessed using self-report questionnaires, which may be subject to social desirability and response bias. Self-reported measures may not fully capture the complexity of team dynamics and cohesion experienced by participants. Future studies could incorporate complementary approaches, such as peer evaluations, observational methods, or behavioral indicators of team cohesion, to triangulate findings and provide a more comprehensive assessment of group cohesion. In several MAES© dimensions, post-intervention scores approached the upper limit of the scale, suggesting a possible ceiling effect that reduced sensitivity to detect additional improvements.

The study evaluated group cohesion only in the short term, immediately following the intervention. Without longitudinal follow-up, the durability of the observed improvements remains uncertain. Future studies should include longer-term follow-up assessments to examine whether these effects are maintained over time. It would be particularly relevant to track group cohesion during clinical placements or at the beginning of professional practice, as these represent critical stages in healthcare training.

The potential influence of instructors was not controlled in this study, nor were potential mediating variables such as motivation, self-efficacy, shared leadership, and psychological safety. These factors could influence the relationship between the learning methodology and group cohesion. Future research should examine these variables to gain deeper insight into the mechanisms underlying the observed associations.

Building on the above limitations, several important directions for future research can be identified. First, longitudinal studies would help to assess the sustainability of the observed effects on group cohesion over time, particularly in real-world healthcare settings. In addition, examining the relationship between group cohesion and relevant clinical outcomes, such as interprofessional communication, error reduction, and patient safety, would provide valuable evidence of educational impact. The role of potential mediating variables, including motivation, self-regulation, and shared leadership, should also be explored to better understand the mechanisms explaining these effects. Further research using randomized controlled multicenter trials would strengthen causal inference and improve the generalizability of findings across educational contexts. Moreover, mixed-methods approaches incorporating qualitative data could provide a more comprehensive understanding of group cohesion dynamics and team development. Finally, evaluating the cost-effectiveness of the MAES© methodology compared with other active learning strategies would be important for informing its feasibility and scalability in educational practice. Collectively, these approaches would contribute to a more robust understanding of how MAES© may be associated with group cohesion and team performance in healthcare education.

## 5. Conclusions

This study adds to the growing evidence on clinical simulation in nursing education by comparing group cohesion outcomes between self-directed (MAES©) and non-self-directed (SBL) approaches. Both groups showed significant pre–post improvements in group cohesion, although MAES© participants achieved higher adjusted post-intervention scores across all assessed dimensions, particularly in task-oriented cohesion (GI-T).

These results suggest that self-directed, collaborative, and autonomy-oriented simulation environments tend to be associated with more favorable group cohesion outcomes in nursing education and healthcare training.

However, the quasi-experimental, non-randomized design, the allocation of participants by university, and baseline differences between groups limit causal inference and likely introduced institutional and contextual confounding. Accordingly, the observed differences should be interpreted as associations rather than effects of the intervention.

Future research should use randomized multicenter designs with students from different academic years, universities, and healthcare disciplines to better understand these associations and further explore the mechanisms underlying group cohesion and related non-technical skills.

## Figures and Tables

**Table 1:** Spanish short version of the Group Environment Questionnaire (GEQ) for nursing students.

Number	Item
1	I like to participate in extracurricular activities with the other members of my group (dinners, excursions …)
2	I am happy with my contributions to the work of the group
3	I have good friends in this group
4	In this group I can perform to the best of my ability
5	Group members are one of the most important social groups to which I belong
6	I like the style of work of this group
7	Group members like to party together
8	Group members join forces to achieve the objectives during the preparation and conduct of the simulation sessions
9	Group members would like to get together a few times after the clinical simulation is over
10	All members take responsibility for a poor group performance
11	Our group members would like to meet in situations other than preparing and conducting simulation sessions
12	If there is a problem during the preparation of the simulation sessions, all members join forces to overcome it

Note: García-Álvarez et al. [19].

**Table 2:** Sociodemographic characteristics of the sample by intervention group.

	MAES©	SBL	Test	χ^2^	*p*-Value
Participants	188	123			
Age (years), mean ± SD	23.67 ± 4.81	23.35 ± 6.03	Mann–Whitney U		0.810
Gender (%)			Chi-square	0.000	0.976
Female	78.19	78.05			
Male	21.81	22.95			
Province of origin (%)			Chi-square	55.889	<0.001
Murcia	56.38	93.94			
Other	43.62	6.06			
Previous academic qualifications (%)			Chi-square	0.137	0.933
None	81.38	82.11			
Non-university	12.77	13.01			
University	5.85	4.88			
Employment status (%)			Chi-square	0.334	0.563
No	92.02	89.43			
Yes	7.98	10.57			

Note. SD = standard deviation; MAES© = experimental group; SBL = control group.

**Table 3:** Within-group pre–post changes in group cohesion dimensions.

	Pre-Intervention				Post-Intervention			
	ATG-S	ATG-T	GI-S	GI-T	ATG-S	ATG-T	GI-S	GI-T
MAES©								
Median	11	12	11	11	14	14	13	14
IQR	4	4	4	4	2	2	4	2
Mean ± SD	10.93 ± 2.56	11.37 ± 2.64	10.61 ± 2.76	10.87 ± 2.77	13.33 ± 1.87	13.35 ± 1.27	12.65 ± 2.53	13.80 ± 1.51
SBL								
Median	10	11	11	11	12	13	12	13
IQR	4	4	3	3	3	3	5	4
Mean ± SD	10.28 ± 2.82	10.75 ± 2.50	10.28 ± 2.22	10.47 ± 2.35	12.13 ± 1.80	12.61 ± 1.81	11.63 ± 2.64	12.30 ± 2.27

Note. Values are presented as median, interquartile range (IQR) and mean ± standard deviation (SD); ATG-S = Individual Attraction to Group—Social; ATG-T = Individual Attraction to Group—Task; GI-S = Group Integration—Social; GI-T = Group Integration—Task; MAES© = experimental group; SBL = control group.

**Table 4:** ANCOVA results for post-intervention group differences adjusted for baseline scores.

Dimension	Adjusted Mean MAES© (95% CI)	Adjusted Mean SBL (95% CI)	Mean Difference (95% CI)	F(df1, df2)	*p*-Value	ηp^2^ (95% CI)
ATG-S	13.30 (13.04–13.57)	12.17 (11.85–12.50)	1.13 (0.71–1.55)	F(1308) = 28.29	<0.001	0.084 (0.034–0.148)
ATG-T	13.35 (13.14–13.57)	12.60 (12.33–12.87)	0.75 (0.40–1.10)	F(1308) = 18.11	<0.001	0.056 (0.016–0.112)
GI-S	12.65 (12.28–13.02)	11.63 (11.17–12.09)	1.02 (0.43–1.61)	F(1308) = 11.59	0.001	0.036 (0.007–0.086)
GI-T	13.81 (13.54–14.07)	12.30 (11.97–12.62)	1.51 (1.09–1.93)	F(1308) = 49.45	<0.001	0.138 (0.075–0.211)

Note. ATG-S = Individual Attraction to Group—Social; ATG-T = Individual Attraction to Group—Task; GI-S = Group Integration—Social; GI-T = Group Integration—Task; MAES© = experimental group (UCAM); SBL = control group (UMU). Adjusted means were estimated using ANCOVA controlling for baseline scores. CI = confidence interval; Mean Difference = adjusted between-group mean difference (MAES© − SBL); F(df1, df2) = ANCOVA F-statistic with numerator and denominator degrees of freedom; ηp^2^ = partial eta-squared effect size.

**Table 5:** Within-group pre–post changes in group cohesion.

	MAES©			SBL		
	Z	*p*-Value	r (95% CI)	Z	*p*-Value	r (95% CI)
ATG-S_1–ATG-S_2	−8.719 *	<0.001	0.636 (0.542–0.714)	−5.909 *	<0.001	0.533 (0.391–0.646)
ATG-T_1–ATG-T_2	−7.557 *	<0.001	0.551 (0.443–0.643)	−5.914 *	<0.001	0.533 (0.391–0.646)
GI-S_1–GI-S_2	−6.485 *	<0.001	0.473 (0.354–0.577)	−4.356 *	<0.001	0.394 (0.233–0.532)
GI-T_1–GI-T_2	−9.694 *	<0.001	0.707 (0.627–0.772)	−5.468 *	<0.001	0.493 (0.342–0.614)
Total Score_1–Total Score_2	−9.772 *	<0.001	0.713 (0.634–0.777)	−7.016 *	<0.001	0.633 (0.512–0.726)

Note: Statistical tests used: Wilcoxon signed-rank test (Z and *p*-value) and Rosenthal’s r effect size with 95% confidence intervals (95% CIs); * Negative ranks indicate higher post-intervention than pre-intervention scores; ATG-S = Individual Attraction to Group—Social; ATG-T = Individual Attraction to Group—Task; GI-S = Group Integration—Social; GI-T = Group Integration—Task.

## Data Availability

The data used to support the findings of this study are available from the corresponding author upon request.

## Public Involvement Statement

No public involvement was present in any aspect of this research.

## Guidelines and Standards Statement

This manuscript was drafted against the Transparent Reporting of Evaluations with Non-Randomized Designs (TREND) guidelines for nonrandomized interventions.
